# Retraction: “Screening of candidate genes and fine mapping of drought tolerance quantitative trait loci on chromosome 4 in rice (*Oryza sativa L*.) under drought stress” by Nie, Y. Y., L. Zhang, Y. H. Wu, H. J. Liu, W. W. Mao, J. Du, et al.

**DOI:** 10.1002/ece3.1855

**Published:** 2015-12-10

## Abstract

The above article, published online on 15 October 2015 in Wiley Online Library (http://onlinelibrary.wiley.com/doi/10.1002/ece3.1786/abstract), has been retracted by agreement between the authors, the journal Editor in Chief, Allen Moore, and John Wiley & Sons Ltd. The authors have requested the retraction of their paper because they mistakenly analyzed the wrong backcross lines. This mistake was only recently discovered and they are now in the process of repeating the experiment using the correct Backcross Introgression Lines. The authors apologize for this mistake.

**Reference:**

Nie, Y. Y., L. Zhang, Y. H. Wu, H. J. Liu, W. W. Mao, J. Du, et al. 2015. Screening of candidate genes and fine mapping of drought tolerance quantitative trait loci on chromosome 4 in rice (*Oryza sativa L*.) under drought stress. Ecology and Evolution 5(21):5007–5015. doi: 10.1002/ece3.1786.

